# Micromachining of High Quality PMN–31%PT Single Crystals for High-Frequency (>20 MHz) Ultrasonic Array Transducer Applications

**DOI:** 10.3390/mi11050512

**Published:** 2020-05-19

**Authors:** Zhihong Lei, Yan Chen, Guisheng Xu, Jinfeng Liu, Maodan Yuan, Lvming Zeng, Xuanrong Ji, Dawei Wu

**Affiliations:** 1 State Key Laboratory of Precision Electronic Manufacturing Technology and Equipment, Guangdong University of Technology, Guangzhou 510006, China; leizhihong-gdut@foxmail.com (Z.L.); yanchen@gdut.edu.cn (Y.C.); mdyuan@gdut.edu.cn (M.Y.); zenglvming@163.com (L.Z.); 2 Key Laboratory of Transparent Opto-Functional Advanced Inorganic Materials, Shanghai Institute of Ceramics, Chinese Academy of Sciences, Shanghai 201899, China; gshxu@mail.sic.ac.cn (G.X.); jinfengliu@mail.sic.ac.cn (J.L.); 3 Key Lab of Optic-Electronic and Communication, Jiangxi Science and Technology Normal University, Nanchang 330038, China; 4 State Key Laboratory of Mechanics and Control of Mechanical Structures, Nanjing University of Aeronautics and Astronautics, Nanjing 210016, China

**Keywords:** laser micromachining, PMN–31%PT single crystals, high-frequency transducer, micron-sized kerf, piezoelectric performance, ferroelectric domain, piezoelectric coefficient

## Abstract

A decrease of piezoelectric properties in the fabrication of ultra-small Pb(Mg_1/3_Nb_2/3_)–*x*%PbTiO3 (PMN–*x*%PT) for high-frequency (>20 MHz) ultrasonic array transducers remains an urgent problem. Here, PMN–31%PT with micron-sized kerfs and high piezoelectric performance was micromachined using a 355 nm laser. We studied the kerf profile as a function of laser parameters, revealing that micron-sized kerfs with designated profiles and fewer micro-cracks can be obtained by optimizing the laser parameters. The domain morphology of micromachined PMN–31%PT was thoroughly analyzed to validate the superior piezoelectric performance maintained near the kerfs. A high piezoresponse of the samples after micromachining was also successfully demonstrated by determining the effective piezoelectric coefficient (d_33_*~1200 pm/V). Our results are promising for fabricating superior PMN–31%PT and other piezoelectric high-frequency (>20 MHz) ultrasonic array transducers.

## 1. Introduction

Pb(Mg_1/3_Nb_2/3_)–*x*%PbTiO_3_ (PMN–*x*%PT) and other relaxor-based ferroelectric crystal materials have drawn extensive interest in the development of high-frequency (>20 MHz) ultrasonic array transducers [1,2,3]. Compared with traditional ceramic materials (i.e., Pb[Zr(*x*)Ti(1–*x*)]O_3_ (PZT), BaTiO_3_ (BT)), ultrasonic transducers with PMN–PT provide outstanding merits, including wider bandwidth (*BW*), higher sensitivity, and higher electromechanical coupling coefficient (*k_t_*), thus achieving high-quality imaging [4,5,6]. Sun et al. designed a 35 MHz PMN–PT 1–3 composite transducer, which provides an insertion loss of 18 dB and an axial resolution of 30 μm with −6 dB bandwidth, as high as 100% [7]. Zhou et al. developed a 50 MHz PMN–PT array with 90% bandwidth, resulting in an insertion loss of 19.5 dB and an axial resolution of 25 μm [8]. Despite the superior performance of PMN–PT high-frequency ultrasonic transducer, micromachining of these materials remains challenging because of their brittleness and low phase-transition temperature. For example, the PMN–PT wafers are known to be particularly prone to chipping and forming micro-cracks during the traditional dicing process [5,9,10]. Such defects would substantially degrade the piezoelectric properties of PMN–PT, considerably reducing the bandwidth and sensitivity of transducers [9,10]. In most studies, high-frequency ultrasonic array transducers using PMN–PT and their composites were developed by low-stress dry and wet chemical etching techniques [7,8,11]. However, the etching processes are complicated, time consuming, very expensive, and pollutional [12,13,14].

Ultrashort pulse laser micromachining, especially for femtosecond and picosecond laser techniques, offers an alternative to etching techniques for micron-size kerf cutting [15,16,17,18]. Compared with etching techniques, laser processing is simple, highly productive, cheap, and environment friendly. Moreover, laser micromachining has been successfully applied to the development of various high-frequency Pb[Zr(*x*)Ti(1–*x*)]O_3_ (PZT) array transducers [19,20,21,22]. With the growing demand of PMN–PT high-frequency transducers, patterning PMN–PT single crystals with micron size and high piezoelectric performance by laser micromachining is of great interest [23]. For high-frequency (>20 MHz) PMN–PT ultrasonic array transducers, kerfs with ultrasmall width (<30 μm) between array elements can effectively avoid formation of grating lobes [24]; the element width must be less than 50 μm to maintain a high longitudinal aspect ratio [5,24], and these small elements with high piezoelectric properties can ensure the wide bandwidth and high sensitivity of transducers [24]. However, the effects of the small (<30 μm) kerfs and piezoelectric properties of PMN–PT based on laser micromachining, which are decisive factors for high-frequency transducers performance evaluation, have not been sufficiently researched.

The ultrahigh piezoelectric properties of PMN–PT single crystals are primarily related to their fine ferroelectric domain structures [25]. Wang et al. used computer modeling and simulation to reveal the mechanism of domain wall broadening, explaining the domain structures effect on the piezoelectric properties of single crystals [26]. Ahluwalia R et al. established the role played by domain walls in nucleating field-induced transition on the basis of the continuum Ginzburg–Landau model. They showed that the piezoelectricity of crystals can be enhanced by small-size domains [27]. Zhang et al. found that the piezoelectric coefficient (*d_33_*) increased significantly from 450 pC/N to 1630 pC/N as the domain size decreased from 50 μm to 500 nm [28]. Therefore, the local piezoelectric performance of the material after micromachining can be evaluated in terms of the domain morphology.

Hence, in this study, a 355 nm Nd:YVO_4_ laser system is proposed to micromachine PMN–31%PT single crystals with micron kerfs and high piezoelectric performance for high-frequency (>20 MHz) array transducers. The relationships between kerf profile and laser processing parameters such as power, repetition frequency, scanning cycles, and speed are determined using an optical microscope (OM). The microstructure of the kerfs was observed using a scanning electron microscope (SEM). To evaluate the local piezoelectric properties, the domain morphology near the kerf is qualitatively characterized by polarized light microscopy (PLM) and piezoresponse force microscopy (PFM). The effective piezoelectric coefficient (d_33_*) is further tested, quantitatively confirming that the high piezoresponse of PMN–31%PT is maintained even after micromachining.

## 2. Materials and Methods 

Focusing ultrashort laser pulses on a material will result in ultrahigh fluence around the focus region in nonlinear multiple photon absorption, which further induces structural changes in the material. These changes occur in an extremely short time and less influence on the regions that are out of focus [29,30]. During laser processing of a material, the quality of the kerf mainly depends on the laser parameters and the thermal characteristics of the material. The incident energy (E), corresponding laser energy density (E(r)), and effective pulses number ($p_{n}$) can be calculated as follows [31,32]:

(1) $$E=\frac{P}{f}$$

(2) $$E(r)=\frac{2P_{avg}}{f\pi \cdot \omega_{0}{}^{2}}e^{-2\frac{r^{2}}{\omega_{0}{}^{2}}}$$

(3) $$p_{n}=k\cdot \frac{\omega_{0}f}{v}$$

where P is the average laser power, f is the repetition frequency, $\omega_{0}$ is the radius of laser beam, r is the distance to the focal point, k is the number of scanning cycles, and v is the scanning speed. In this study, a 355 nm Nd:YVO_4_ laser system (LPKF ProtoLaser U3, LPKF Laser & Electronics, Garbsen, Germany) was employed to study the kerf profile and piezoelectric properties of PMN–31%PT after micromachining; the laser parameters are listed in Table 1.

**Figure 1:**
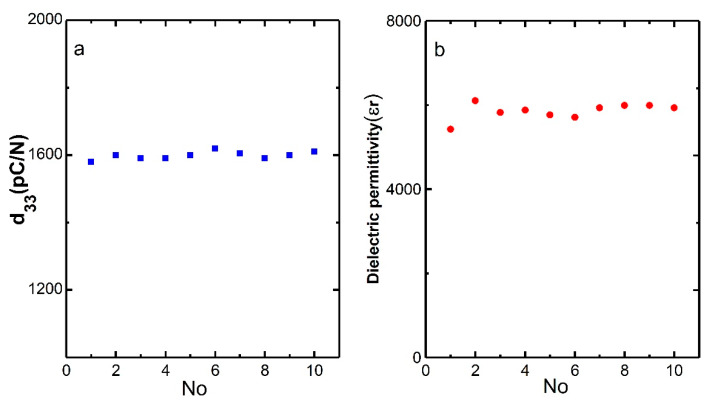
Piezoelectric coefficient d_33_ (**a**) and dielectric permittivity (**b**) of the Pb(Mg_1/3_Nb_2/3_)–*x*%PbTiO_3_ (PMN–PT) samples.

The PMN–31%PT single crystal with a [001] orientation (Innovia Materials Co., Ltd., Shanghai, China) was grown by the vertical gradient freeze (VGF) method [33]. Before laser micromachining, PMN–PT crystals were diced into 10 × 10 mm^2^ at a rate of 3 mm/s by ADT7100 (Advanced Dicing Technology Ltd., Yokneam, Israel) and polished to 0.5 mm thickness. The material performance was tested according to the IEEE Standard on Piezoelectricity 176–1987 using an impedance analyzer (Agilent-Keysight E4490A, Colorado Springs, CA, USA) at 25 °C for 24 h after poling by 150 V. The poled PMN–31%PT single crystals with ultrahigh piezoelectric constant (~1600 pC/N) and dielectric permittivity (~6000) are shown in Figure 1a,b respectively.

The PMN–31%PT crystal was scanned using a UV laser along a 2 mm long line in every trial, and each test was conducted two times. A constant effective focus spot of size 20 μm was focused on the sample surfaces for the first scanning cycle. The power, repetition frequency, speed, and scanning cycle of laser processing parameters were analyzed. The surface and geometry of the kerf were examined using an OM (VHX-1000E, KEYENCE Co., Ltd., Osaka, Japan) with 500 times magnification. All data were obtained as average values of the two readings. The microstructure of kerf was also observed using a scanning electron microscope (SEM, VEGA3, TESCAN Co., Ltd., Brno-Kohoutovice, Czech Republic) with 1000 times magnification.

Two powerful tools, polarized light microscopy (PLM, BX51M, Olympus Co., Ltd., Tokyo, Japan) and piezoresponse force microscopy (PFM, Cypher ES, Asylum Research Co., Ltd., Oxford, UK), were used to investigate the domain structure after laser micromachining. In PLM experiments, the magnification of the microscope was adjusted to 400 times. In PFM experiments, the microdomain images of size 3 × 3 μm^2^ were studied at a fixed distance of ~10 μm from the kerf. Conductive silver (FP16034, Ted Pella Inc., Redding, CA, USA) was coated on one side of the crystals to form the bottom electrode. An AC modulating voltage of 2.0 V (peak-to-peak) was applied through the conductive probe (ASYELEC.01-R2 (*k* = 2.8 nN/nm)) onto the PMN–31%PT surface. To confirm the extent of ferroelectricity and piezoresponse of PMN–31%PT, a sequence of DC bias imposed by 0.1 V AC driving voltage was applied to acquire the strain-electric field hysteresis loop and butterfly loop as well as to further evaluate the effective piezoelectric coefficient (d_33_^*^).

## 3. Results and Discussion

### 3.1. Geometry of Kerf

**Figure 2:**
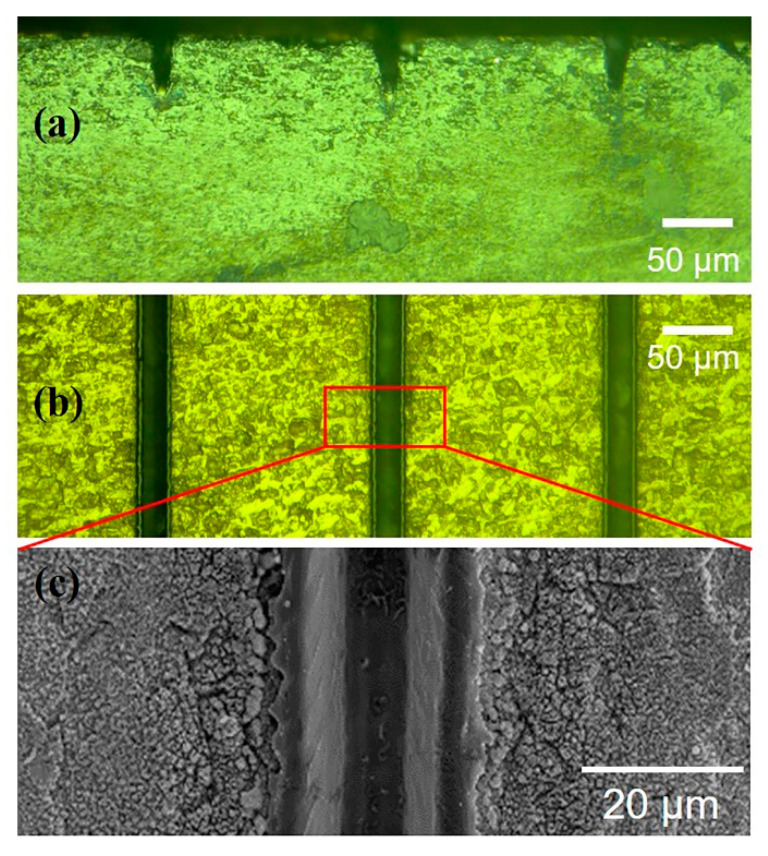
Optical microscope images (**a**,**b**) and scanning electron microscope image (**c**) of laser micromachined PMN–31%PT under 1.0 W, 50 kHz, 500 mm/s, and 20 scanning cycles.

Figure 2a,b show the surface and geometry of the kerf after laser processing (1.0 W, 50 kHz, 500 mm/s, and 20 scanning cycles), respectively. After micromachining, the kerfs are elliptical paraboloid (Figure 2a) owing to the Gaussian function distribution of laser energy [31]. Clear and uniform kerfs with a narrow width (~20 μm) are observed, as shown in Figure 2b. The narrow kerf also shows less micro-cracks and relatively low roughness in the SEM image (Figure 2c). As the results show, in comparison with the damages caused by mechanical dicing [9], laser micromachining has greater potential for retaining the ultrahigh piezoelectric performance of the micron-size single crystals of PMN–31%PT.

**Figure 3:**
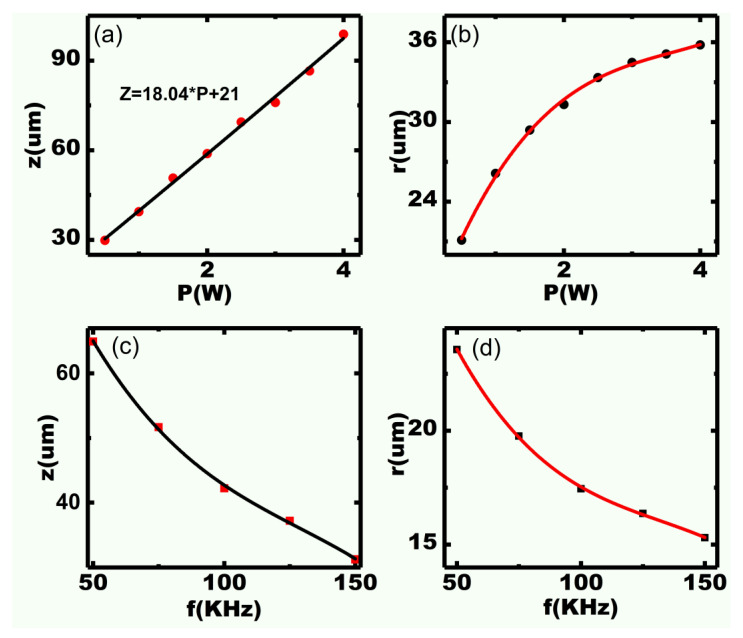
Variation of laser power (**a**,**b**) and frequency (**c**,**d**) with respect to ablated depth (*z*) and width (*r*).

Figure 3a,b show the relationship of power with ablated depth (*z*) and width (*r*), respectively. In the experiments, we set 50 kHz, 500 mm/s, and 20 scanning cycles with various power values. We note that the ablated depth (*z*) and width (*r*) increase with power. The depth (*z*) is proportional to power because the incident energy is directly proportional to power according to Equation (1). Unlike the ablated depth, the width (*r*) increases significantly at a low power (<1.5 W) and changes slightly under a higher power, as shown in Figure 3b. The largest width of the kerf is less than 36 μm. This is mainly because the corresponding laser energy density decreases in the Gaussian distribution, according to Equation (2), and most absorbed energy is changed to heat energy in vapor. The results suggest that deeper and relatively narrower kerfs can be obtained by processing under high power.

Figure 3c,d show the dependence of repetition frequency on ablated depth (*z*) and width (*r*), respectively. Different frequencies (50–150 kHz) under 0.5 W, 500 mm/s, and 50 scanning cycles were applied for the experimental design. The ablated width (*r*) and depth (*z*) decrease with increasing frequency because the incident laser energy is inversely proportional to frequency according to Equation (1). Furthermore, smaller energy (3.33 μJ according to Equation (1)) can be achieved by large frequency (150 kHz), and a small width (~15 μm) can be obtained successfully, as shown in Figure 3d. A small kerf is crucial for realizing high-frequency (>20 MHz) array transducers, which can effectively avoid the grating lobe [34].

**Figure 4:**
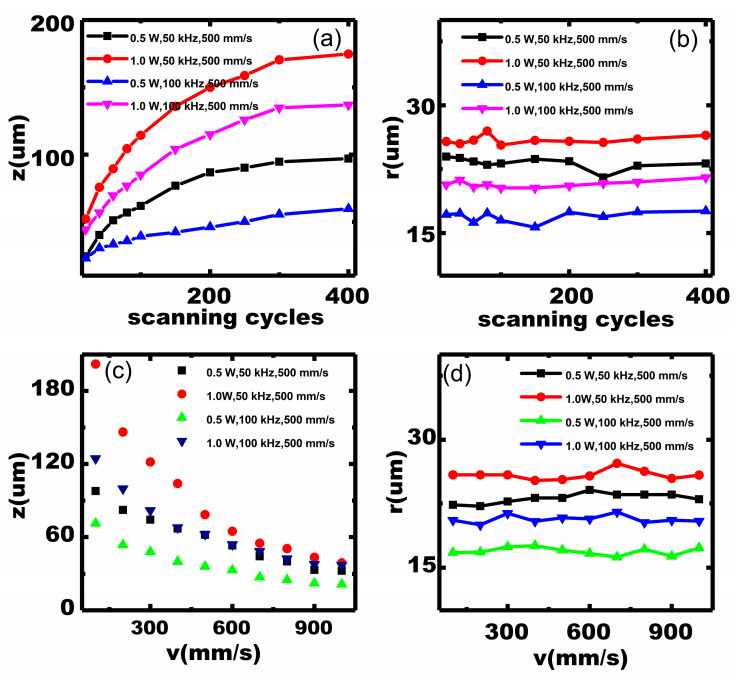
Dependence of the number of scanning cycles (**a**,**b**) and speed (**c**,**d**) on ablated depth (*z*) and width (*r*).

Because the ablated depth (*z*) is limited at a small number of scanning cycles, multiple scanning cycles could be selected to produce a deeper kerf by laser micromachining. Figure 4a,b show the results of the ablated depth (*z*) and width (*r*) as a function of scanning cycles, respectively. We mainly observe that the depth increases with the number of scanning cycles because of the increasing accumulated thermal effect with more cycles. Nevertheless, this relationship is not linearly proportional. In other words, when the number of scanning cycles is small, the depth increases considerably with scanning cycles, whereas the increase in depth is gradual after a certain number of cycles. This is because the laser beam offset in a cycle is different from that in the preceding one, and the laser energy reduces with scanning cycles, as predicted by Equation (2).

Figure 4c,d show the dependence of scanning speed on depth (*z*) and width (*r*), respectively. We note that the ablated depth is inversely proportional to the scanning speed and the largest depth–width ratio reaches 8. This is because, according to Equation (3), fewer pulses in each cycle are used at a high speed [32]. Moreover, the ablated width remains almost constant (<3 μm) with different scanning cycles and speed, as shown in Figure 4b,d. This implies that scanning cycles and speed affect the width to a small extent, mainly because the laser energy varies with the power and repetition frequency, according to Equations (1) and (2), respectively. The results indicate that kerfs with a larger depth–width ratio can be achieved by higher scanning cycles and a lower speed.

The effect of the 355 nm laser on the kerf profile of micromachined PMN–31%PT single crystals was studied systematically. The micron kerf is clean with well-defined edges and fewer extended micro-cracks. The ablated depth is proportional to power and increases slightly after a certain number of scanning cycles; the ablated width decreases with the repetition frequency. Therefore, the scanning cycles and speed affect the ablated width to a certain extent, because the incident energy varies with power and repetition frequency. The above-mentioned relationships provide references to process designated kerfs. Finally, the smallest width of 15 μm and the largest depth–width ratio of 8 were obtained.

### 3.2. Piezoelectric Characterization

**Figure 5:**
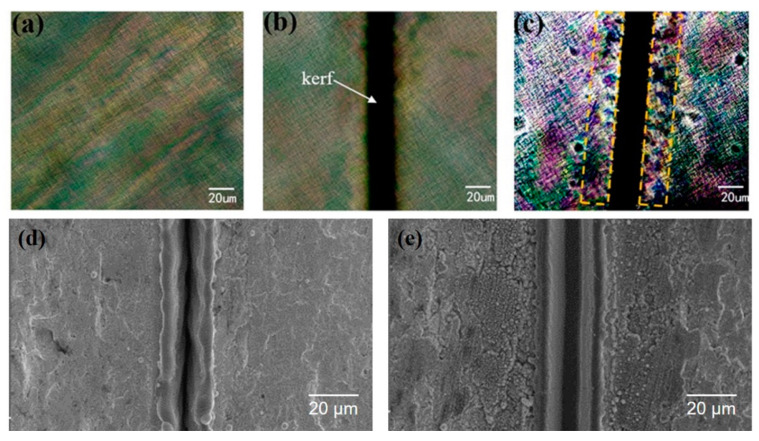
Polarized light microscope images of ferroelectric domains for the PMN–31%PT micromachined at different powers (**a**–**c**) and the scanning electron microscope images of micromachined kerfs (**d**,**e**). Initial domain morphology of the sample without micromachining (**a**) and those micromachined at 0.5 W (**b**,**d**) and 1.0 W (**c**,**e**).

We characterized the domain morphology to evaluate the piezoelectric properties of the micromachined PMN–31%PT. Figure 5b,c show the PLM images of PMN–31%PT after micromachining with different power values (0.5 W and 1.0 W) under 50 kHz, 500 mm/s, and 50 scanning cycles. We compare the domain structures of PMN–31%PT micromachined at 0.5 W and those without micromachining. Clearly, the domains appear in the form of narrow (<1 μm) contrast lines, as shown in Figure 5a; the same is observed near the kerf, as shown in Figure 5b. The domain morphology obtained in this study is consistent with recent reports [3,25], illustrating that laser micromachining has a minimal effect on the piezoelectric properties of the materials. According to the study by Yao, the dark regions in the PLM images mainly form owing to the disappearance of domains, which means that depolarization occurred in PMN–PT [25]. The affected regions can be distinguished clearly near the kerf (<20 μm) in Figure 5c. The microstructures near the kerfs of PMN–31%PT micromachined at 0.5 W and 1.0 W are also shown in Figure 5d,e, respectively. In Figure 5e, clearly, the microstructure of PMN–31%PT is smaller and the affected zone is less than 20 μm. The above results reveal that laser energy (<20 μJ according to Equation (1)) should be controlled to ensure that laser micromachining does not considerably affect the piezoelectric properties.

**Figure 6:**
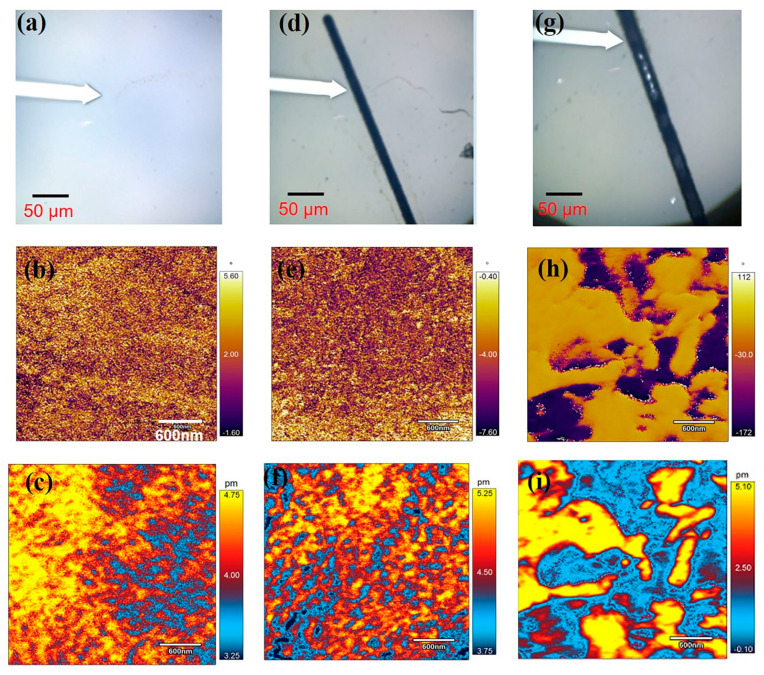
Schematic of the experiment conducted using the piezoresponse force microscope (PFM) (**a**,**d**,**g**), PFM phase (**b**,**e**,**h**), and amplitude (**c**,**f**,**i**) images of ferroelectric domains. The sample without micromachining (**a**–**c**), and those micromachined at 0.5 W (**d**–**f**) and 1.0 W (**g**–**i**).

To evaluate the piezoelectric properties of laser effects more specifically, the local microdomain near the kerf (~10 μm) was also investigated by PFM. The PFM phase and amplitude images are shown in Figure 6b,e,h and c,f,i, respectively. The color in the phase images (Figure 6b,e) is virtually uniform, suggesting that the polarized domains are in the same direction. In addition, as shown in Figure 6c, the speckle-shaped nanometer-sized (<100 nm) domains are compact and uniform. The domain wall motion can occur easily owing to the smaller domains, thus revealing the high piezoelectric activity of the PMN–PT samples [28,35]. In comparison, the domain structure near the kerf, as shown in Figure 6f, is similar to that in Figure 6c. This also confirms that laser micromachining has a minimal effect on the domain morphology. Note that the domain direction changes locally and the domain size (>200 nm) increases sharply in Figure 6h,i, respectively. In addition, the average PFM amplitude in Figure 6i is less than 2.5 pm. As R. Shrout reported, the piezoelectric properties of PMN–PT decrease with increasing domain size. The experimental results suggest that the effect during laser ablation changes the domain characteristics, resulting in decreasing piezoelectric performance. These results also reveal that laser micromachining of PMN–31%PT at optimized parameters has a minimal effect on the microdomain near the kerf (<10 μm) and shows great improvement compared with what is reported in recent studies [36].

**Figure 7:**
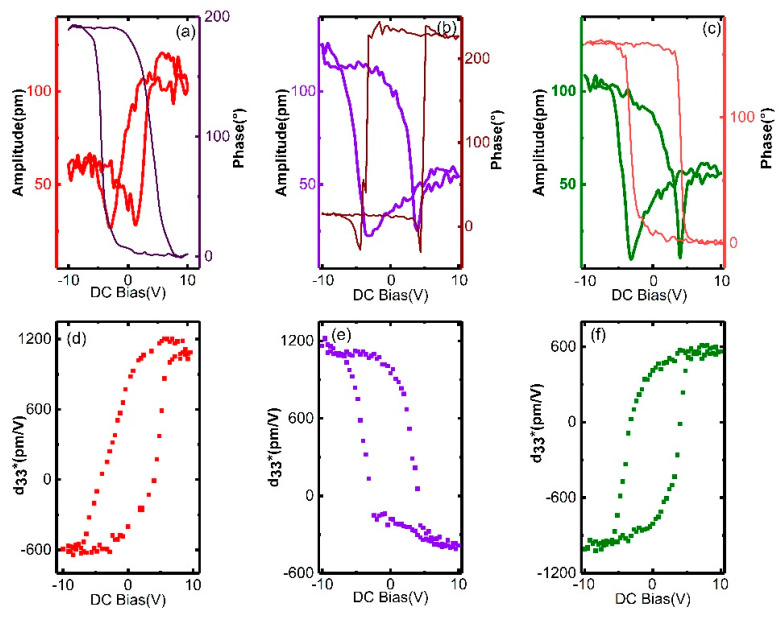
Amplitude–voltage butterfly loops, phase–voltage hysteresis loops, and piezoelectric hysteresis loops of PMN–31%PT samples. The sample without micromachining (**a**,**d**), and those micromachined at 0.5 W (**b**,**e**) and 1.0 W (**c**,**f**).

To further validate the low effects of laser micromachining, we studied the local ferroelectricity near the kerf (~10 μm) by switching the PFM mode. A DC bias up to 10 V was used and the piezoresponse was measured by an AC modulation voltage of 0.1 V. The PFM phase–voltage hysteresis and amplitude–voltage butterfly loops are shown in Figure 7a–c. All hysteresis loops reveal domain switching without leakage behavior. In addition, as shown in Figure 7d–f, d_33_* is acquired by A⋅cos(θ)/V [37], where A is the PFM amplitude, θ is the phase, and V is the amplitude of AC modulation. For the samples that are processed at a small power, a large d_33_* of ~1200 pm/V is observed in Figure 7d,e. Remarkably, d_33_* reaches up to 1000 pm/V at −10 V DC bias after 1.0 W processing, as shown in Figure 7f. This d_33_* value is twice that obtained in a recent study [37]. The experimental data quantitatively demonstrate that high piezoresponse is maintained even after micromachining.

The piezoelectric properties of PMN–31%PT after laser micromachining were studied qualitatively and quantitatively using the PLM and PFM. After optimizing the laser parameters, the domain structures along the kerf agree well with those of the unprocessed samples; a high piezoresponse (~1200 pm/V) is also observed near the kerfs (~10 μm). These results imply that the ultrahigh piezoelectric properties of the materials are still maintained after micromachining into ultrasmall elements (<50 μm). The results are promising for developing superior PMN–31%PT high-frequency (>20 MHz) ultrasonic array transducers, in which large piezoelectric property reduction in the fabrication of small elements occurs, which is an urgent problem [9,38].

## 4. Conclusions

In this study, micromachining of PMN–31%PT single crystals with micron-sized kerfs and high piezoelectric properties for high-frequency ultrasonic array transducer applications was demonstrated. The effect of the 355 nm laser on the kerf profile of micromachined PMN–31%PT single crystals was determined to provide references to obtain designated kerfs. The micron-sized kerf was clean with well-defined edges and less extended micro-cracks; the smallest width of 15 μm and the largest depth–width ratio of 8 were obtained. The high-quality small kerfs of PMN–31%PT could effectively avoid the grating lobes in the high-frequency transducer design. Meanwhile, the superior piezoelectric properties near the kerfs of PMN–31%PT after micromachining were verified qualitatively and quantitatively. The domain structures along the kerfs were aligned well with those of the samples without processing; high piezoresponse (~1200 pm/V) was also measured near the kerfs (~10 μm). These results suggest that ultrahigh piezoelectric properties of PMN–31%PT were still maintained after micromachining into ultrasmall elements, thereby guaranteeing a wide bandwidth and high sensitivity of high-frequency transducers. The results present new micromachining guidance for developing superior PMN–PT ultrasonic array transducers (>20 MHz) that can solve the challenging issue of large reduction in piezoelectric properties. Moreover, the principles described in this study can be extended to the development of other piezoelectric microdevices.

## Figures and Tables

**Table 1:** Parameters of the 355 nm Nd:YVO_4_ laser system.

Parameters	Specifications
Laser type	Nd:YVO_4_
Laser power	5 W
Laser wavelength	355 nm
Laser pulse frequency	25–200 kHz
Focused beam diameter	20 ± 2 μm

## Abbreviations

The following abbreviations are used in this manuscript:

PMN–PT	Pb(Mg_1/3_Nb_2/3_)–PbTiO_3_
PZT	Pb[Zr(x)Ti(1-x)]O_3_
BT	BaTiO_3_
OM	Optical microscope
SEM	Scanning electron microscope
PLM	Polarized light microscope
PFM	Piezoresponse force microscopy
